# Current Status of Traditional Korean Medicine Services in Public Sector: A Study for Integrating Traditional Korean Medicine into Community Care System

**DOI:** 10.3390/healthcare9050493

**Published:** 2021-04-22

**Authors:** Soo-Hyun Sung, Minjung Park, Jihye Kim, Sun-Woo Jeon, Angela Dong-Min Sung, Eun-Jin Lee, Danny Oh, Jung-Youn Park, Jang-Kyung Park, Kyeong Han Kim

**Affiliations:** 1 Department of Policy Development, National Development Institute of Korean Medicine, Seoul 04554, Korea; koyote10010@nikom.or.kr (S.-H.S.); angelasung84@nikom.or.kr (A.D.-M.S.); eunjin6434@nikom.or.kr (E.-J.L.); 2 National Agency for Development of Innovative technologies in Korean Medicine, Seoul 04554, Korea; mj.park@nikom.or.kr; 3 Research Institute of Korean Medicine Policy, The Association of Korean Medicine, Seoul 07525, Korea; jihyekim1217@gmail.com; 4 Chung-Yeon Central Institute, Gwangju 61949, Korea; oddibbo@naver.com; 5 Graduate School of Social Welfare, Soongsil University, Seoul 06978, Korea; ziseung@gmail.com; 6 Department of Health and Welfare, Yuhan University, Bucheon 14780, Korea; park0625@yuhan.ac.kr; 7 Division of Clinical Medicine, School of Korean Medicine, Pusan National University, Yangsan 50612, Korea; 8 Department of Preventive Medicine, College of Korean Medicine, Woosuk University, Wanju 55338, Korea

**Keywords:** traditional Korean medicine, community care, public service

## Abstract

Korea is currently executing a pilot program for community care of its aging population and aims to implement community care systems on a national scale by 2025. This study examines the traditional Korean medicine (TKM) service to be provided within community care by understanding the current status of TKM services. The Ministry of Health and Welfare (MoHW) sent official letters to 242 local governments (cities, districts, and counties) from October to November 2019 to survey the status of the public TKM services provided in 2018. The items of the survey included basic demographic information as well as information that could reveal how the program was implemented. In 112 local government jurisdictions (response rate 46.3%), a total of 867 TKM service programs were in place. As a result of the survey, it was revealed that they did not have any service manuals or evaluation results. To provide home-care-based TKM service for the elderly as an integrated part of a community care system, it is necessary to develop, distribute, and evaluate a standard service manual including an evaluation index by the central government.

## 1. Introduction

With the development of medical technology, human life expectancy has increased dramatically, thereby resulting in a worldwide trend of an aging population [1,2]. With a rapidly aging population, the present infrastructure for healthcare will prove even more inadequate to meet the physical and mental health needs of senior citizens [2]. Older adults consistently prefer aging in one place, which requires a high level of community support and services that are currently lacking [2,3].

As a solution to this issue of aging, many countries are implementing community care programs that provide services focused on local communities [4]. Community care, which is understood as home care, is a community-led social service policy that provides integrated support covering healthcare, housing, care, recuperation, and independent living so that an individual may receive services that fit their needs, and they can live as a member of the local community [5]. The United Kingdom introduced the Community Care Act in 1990, requiring local governments to undertake the roles and costs for social security so that such governments play the role of caregivers [6,7]. In 2012, the Japanese government implemented a community-based integrated care system that provides seamless community healthcare resources for elderly people with chronic diseases and disabilities [8]. The Canadian government started the home and community care program in 1999, while the federal government established the master plan for home and community care (2013–2023) so that local governments can provide home care and community service for people with disabilities and the elderly [9]. The care or recovery after certain diseases or conditions types of community care include palliative care [10], stroke rehabilitation [11], dementia [12], multiple chronic conditions [13], hip fracture [14] and sarcopenia [15].

In 2017, South Korea entered an aged society in which 14% of the population is elderly, i.e., 65 or older [16]. By 2026, it is expected to become an ultra-aged society, with 20% of its total population being in the elderly category [16]. The Ministry of Health and Welfare (MoHW) announced its pilot programs for community care in 16 local jurisdictions in 2019 [17]. The MoHW is currently working to establish community care systems at the local government level in 242 local jurisdictions by the year 2025, before South Korea becomes an ultra-aged society.

Traditional Korean medicine (TKM) has been used for preventing and treating diseases for a long time. [18] Since the introduction of Western medicine in the 19th century, TKM has been in charge of the Korean medical system along with Western medicine [19]. Traditional Korean medicine (TKM) services are provided in both the public sector and the private sector. In the public sector, the subjects are provided with TKM services free of charge, mainly at community health centers or TKM clinics. Public TKM services are provided to people in forms of health promotion services such as life cycle programs, and disease management services including infertility and dementia treatment [20]. TKM services in the private sector are provided differently, according to the demands of the residents. Currently, TKM practitioners use acupuncture, pharmacopuncture, herbal medicine, cupping, chuna, moxibustion, and other forms of intervention for treating conditions (e.g., disc-related disease, osteoarthritis, shoulder pain, back pain, sprain) [21,22]. Local governments provide various forms of TKM services in their own way [23]. According to the government’s community care implementation policy, the agenda of the fourth master plan for TKM promotion (2021–2025) was set up by the MoHW to provide TKM services within the boundary of community care [23].

This is the first study to survey the current status of TKM services provided in the public local community domain under the initiative of the South Korean government. This study will be used as the basic data for designing TKM policies in government, which will be linked and converged with other welfare programs within the domain of community care. These programs will aim at examining how such TKM services should be provided in the domain of community care by examining the current status of TKM services provided in the public sector. Furthermore, these findings will be of use in establishing the government’s policy to review the utilization and introduction of TKM in community care.

## 2. Materials and Methods

### 2.1. Scope of the Survey

The samples of this study were composed of 242 localities (cities, districts, and counties) over the entire territory of South Korea.

### 2.2. Development of the Survey Form

The draft of the survey form was developed by three researchers (S.-H.S., A.D.-M.S.., and E.-J.L.) based on preceding studies [24,25] and was reviewed and revised by three other researchers (D.O., J.-K.P., and K.H.K.). The six researchers who developed and revised the forms jointly discussed and agreed upon the final version of the form. The form was composed of six items covering the basic information on programs and another six items on the form of the program. The basic information (the name of the program, targets, contents, number of potential subjects, budget, and organization that provided the services) was to be provided using a form that was distributed free of cost, while the participants could answer either “yes” or “no” for each question regarding the programs (visit/outpatient programs, existence of outcomes of the program, satisfaction level survey, performance of evaluation, existence of a program manual, presence of training or consultation materials). The “don’t know” answer option was not considered due to the characteristics of official government investigation.

### 2.3. Survey Method

The MoHW conducted the survey of the current status in 242 localities (cities, counties, and districts) across South Korea by sending official letters via their electronic document system over the period from 1 October to 31 October 2019, to survey the current status of the TKM services implemented in 2018. The questionnaire forms were filled out by the persons in charge of TKM-related programs within the relevant local governments. The researchers did not request replies if there were no TKM-related programs.

In addition to asking the participants to complete the questionnaire forms, the researchers also requested that the participants submit additional documents or materials (e.g., program outcome report, satisfaction survey results, evaluation results, program manual, and training/consultation materials). The researchers reviewed the questionnaires recovered by the MoHW and requested the organizations that submitted the questionnaire forms to provide supplementary materials in the case of any missing data.

### 2.4. Data Classification and Analysis

Four researchers (S.-H.S., M.P., J.-K.P., and K.H.K.) established the criteria for classifying the data gathered by the types of programs. First, the researchers classified the programs into health promotion types, TKM treatment types, complex types (health promotion plus TKM treatment), and awareness improvement types. Then, these categories were further sub-divided based on the target populations (e.g., pediatric and juvenile, adults, women, disabled, or elderly). If it was difficult to sub-categorize, the categories were sub-divided based on the types of programs. In addition, the researchers selected key examples of the first four categories and summarized the content of the TKM services they provided.

## 3. Results

### 3.1. Demographic Characteristics of the Respondent

A total of 112 questionnaires were collected, which means that responses were obtained from each of the 112 localities in South Korea (response rate 46.3%) (Table 1).

### 3.2. The Current Status of All of the TKM Services Provided in Local Communities

A total of 112 localities (46.3%) were providing 867 TKM services, and the characteristics of each type are as shown in Table 2. Of the 867 TKM services, the complex type accounted for 319 (36.8%), being the largest in number. This was followed by the TKM treatment type, accounting for 295 (34.0%), health promotion type (*n* = 219, 25.3%), and awareness improvement type (*n* = 34, 3.9%).

Of the 867 services provided by local communities, 751 (86.6%) were being provided to the organizations that provided services to the subjects who visited them or came as outpatients (TKM clinics, community health centers, etc.). The remaining 116 (13.4%) were providing services by visiting the residents or public facilities used by the subjects; 203 programs had program execution reports (23.4%), while 171 (19.7%) conducted customer satisfaction level surveys. Eight (0.9%) programs conducted evaluations, while 22 (2.5%) had program manuals; 15 (1.7%) had training and consultation materials.

#### 3.2.1. Health Promotion Services Type of TKM Public Services

Of the 219 programs of health promotion type, programs intended for the elderly were the largest in number (*n* = 170, 77.6%), followed by programs for adults (*n* = 43, 19.7%), the disabled (*n* = 4, 1.8%), and pediatrics and juveniles (*n* = 2, 0.9%) (Table 2). The key examples of the health promotion programs intended for the elderly are shown in Table 3.

#### 3.2.2. Complex Service Type (TKM Treatment Plus Health Promotion) of TKM Public Services

Of the 319 complex service type programs, programs for women were the largest in number (*n* = 162, 50.8%), followed by programs for pediatrics and juveniles (*n* = 83, 26.0%), the elderly (*n* = 33, 10.3%), adults (*n* = 27. 8.5%), and the disabled (*n* = 14, 4.4%). Key examples of the complex service types intended for women are shown in Table 3.

#### 3.2.3. TKM Treatment Services Type of TKM Public Services

Of the 295 TKM treatment service types, general treatment types were of the largest number (*n* = 231, 78.3%), followed by outpatient care (*n* = 51, 17.3%); 13 (4.4%) were family doctor type services. Of the TKM treatment types, key examples of outpatient care are shown in Table 3.

#### 3.2.4. Awareness Improvement Service Type of the TKM Public Services

Of the 34 awareness improvement type services, anti-smoking programs were the largest in number (*n* = 25, 73.5%), followed by TKM experiential programs (*n* = 6, 17.7%) and school TKM programs (*n* = 3, 8.8%). Key examples of the anti-smoking programs in the awareness improvement category are shown in Table 3.

## 4. Discussion

This study was conducted as the first of its kind initiated by the government to understand the current status of TKM service provision in the public sector. The scope of this survey included the entirety of 242 local governments (response rate 46.3%). The organizations that provided TKM services in the public sector were mainly community health centers and primary healthcare providers [23]. The TKM services provided at community health services were revised three times, according to the government’s policy (the master plan for health promotion). They are currently running programs for disease prevention and health promotion [26]. Primary healthcare providers provide services centered around treatment, education, and consultation. However, these programs are implemented separately, depending on the policies and budgets of local governments [23]. For this, the government conducted a survey to understand the current status of the entire TKM services provided in the public sector to shape the policies for providing TKM services in community care, which is to be introduced later.

Of the total 242 localities in the country, 112 (46.3%) provided public TKM services. Of these 867 services, 203 (23.4%) were intended for the elderly and of these elderly programs, 30 (14.8%) were visit types. This indicates that TKM is appropriately integrated into the national system and there are demands for these services among the general population. However, it also shows that the core objective of community care or home care programs for the elderly is not a common practice in TKM public services. In the future, the government endeavors to introduce a policy that allows home care for the elderly. Such a policy is necessary, specifically to meet the needs of the elderly with limited mobility (e.g., patients with severe diseases or those with limited abilities to move) [27].

Of the 203 TKM services intended for the elderly population, none had a service provision manual. In addition, none of these programs were being evaluated by an evaluation index. The South Korean population is one of the fastest aging populations in the world, and it is expected that the country will have the highest ratio of elderly individuals in 2045 [28]. As a solution for this issue, the MoHW has implemented community care pilot programs in 16 localities since 2019. The pilot programs are in effect for the elderly in 13 localities, the disabled in 2 localities, and mental health patients in 1 locality. These programs are focused on the establishment of community care for the elderly. Furthermore, to implement TKM services in the 13 localities where the pilot community care programs for the elderly are in effect, it is necessary to develop a manual for providing the TKM service, including a system for evaluation. Moreover, it is necessary to provide training programs by TKM practitioners to train experts who can apply the developed manual to the treatment.

Of the 867 service programs, 162 were public TKM services for women (18.7%). Of these, 97 were related to childbirth (59.9%). The total childbirth rate of South Korea has been decreasing since 2001 (1.31) and it reached 0.97 in 2018, which was the lowest globally [29,30]. To cope with this issue of low birthrate, together, the government has established a master plan every five years to support medical assistance for childbirth to infertile couples, social protection for childbirth, childcare services, balance of workplace and family, tax incentives, and child education [31]. As such, it can be said that the government’s efforts to solve the severe challenges of sub-fertility and low birthrate that South Korea now faces have been reflected in the process of providing the public TKM services that are related to childbirth. If a low birthrate persists and the elderly population increases, the government’s burden for caring for the elderly will only increase [32]. Therefore, it would be necessary to implement a community care system to manage elderly care and low birthrate together for the policy to be effective. The community of TKM practitioners should be prepared by means of establishing a public service model related to a low birthrate and a corresponding manual.

The TKM services provided by the public sector to the disabled accounted for 2.1% of the total services. While the government is implementing a pilot project of community care for the disabled, a new model for providing a public TKM service for the disabled must be developed. TKM has advantages in treating musculoskeletal diseases and other chronic diseases [33,34]. Furthermore, it is highly preferred and popular among the general public [33,34]. Therefore, the development of models and manuals for managing chronic musculoskeletal diseases through TKM services that can be applied to pilot projects of community care for the disabled is urgently required.

Korean-style community care aims to provide integrated services to the people in need of care, centered around the community in order to overcome the issues of excessive demand for larger hospitals, aging populations, and care mainly provided by public facilities [35]. It was also suggested that the services be focused on linking and integrating healthcare and welfare services [35]. This indicates that while it is important for the government to intervene in the development of policies that ensure proper integration within community care by standardizing TKM services, it is also necessary for the government to intervene regarding the kind of services or job types that can be linked or integrated with the TKM services at the center.

The limitations of this review are as follows: First, this study is a trustworthy survey conducted by the government and is believed to have high accuracy and reliability in terms of the data gathered. However, the limitation is that the survey form’s creditability and validity are yet to be verified. Second, out of the data gathered by local governments, it was very difficult to find any case with program manuals, evaluation results, and training/consultation, which made it very difficult to provide a standard model. In the future, it is necessary to develop, distribute, and evaluate a standard service manual including an evaluation index by the central government. Third, since some of the local governments did not reply to this survey and are still providing TKM services, it is difficult to generalize the findings of this review to the entirety of TKM services. Fourth, it was difficult to evaluate the quality of the program because only quantitative research was conducted. It will be necessary to develop a qualitative survey form to evaluate the quality of the program in the future.

To integrate the TKM services in community care that are to be established across South Korea by 2026, the following issues must be considered in the development of the related policies: First, it should be possible to provide a certain level of standardized TKM services by developing a standard model of TKM for the elderly and develop/distribute corresponding manuals. Second, it is necessary to run a pilot program covered by the national health insurance based on the standard model and manual for the elderly, to establish the infrastructure used to provide TKM services on a continued basis. Third, it is necessary to develop a welfare service linkage model centered around the subjects of TKM services. Such a model must be applied to the 15 pilot TKM programs to create an example of efficient convergence between TKM and welfare services. Lastly, it is necessary to establish a system that can investigate, evaluate, and spread the current status of TKM services by the central government.

## 5. Conclusions

As of 2018, public TKM services were being provided in about half of the localities (46.5%) in South Korea. However, those intended for the elderly accounted for only 23.4%. In addition, 14.8% of these services were outpatient types and did not have service manuals or evaluation results. In the future, it is necessary to develop an outpatient treatment manual, create cases for national expansion to apply for community care pilot programs in localities, and establish a system for evaluation and expansion by the central government so that TKM services based on home care for the elderly can be provided as a part of community care.

## Figures and Tables

**Table 1:** The response by local governments regarding the TKM services provided by communities.

Areas	Total	Response	Percentage (%)
Seoul	25	5	20.0
Gyeonggi	42.	20	47.6
Incheon	10.	3	30.0
Chungbuk	14.	4	28.6
Chungnam	15	10	66.7
Sejong	1	1	100.0
Gangwon	18.	5	27.8
Jeonbuk	14	12	85.7
Jeonnam	22	18	81.8
Gwangju	5	2	40.0
Gyeongbuk	23	13	56.5
Gyeongnam	22.	14	63.6
Daegu	8	1	12.5
Ulsan	5.	1	20.0
Busan	16	2	12.5
Jeju	2	1	50.0
Total	242	112	46.3

**Table 2:** Types of TKM services provided by communities.

Types			No. of Programs (N, %)	Outpatient/ Visit	Program Results	Satisfaction Survey	Evaluation	Program Manuals	Training Materials
Provided Service Types (N, %)	Types of Clients/Sub-Categories								
Health promotion services (219, 25.3%)	Adults		43 (19.7%)	33/10	9	11	0	0	0
	Elderly		170 (77.6%)	142/28	27.	32	0	0	0
	Disabled		4 (1.8%)	0/4	2.	0	0	0	0
	Pediatrics and juveniles		2 (0.9%)	0/2	2	0	0	0	0
Complex services (TKM treatment + health promotion) (319, 36.8%)	Females	Pregnant women	29 (9.1%)	26/3	21	5	0	0	3
		Sub-fertility	68 (21.3%)	68/0	43	15	7	14	0
		Menstrual pain	19 (6.0%)	18/1	6	1	0	1	1
		Climacterium	46 (14.4%)	46/0	7	8	0.	1	0
	Pediatrics and juvenile		83 (26.0%)	81/2	17	1	0	0	3
	Adults		27 (8.5%)	27/0	2	2	0	0	0
	Disabled		14 (4.4%)	13/1	1	2	0	0	0
	Elderly		33 (10.3%)	31/2	11	4	0	0	1
TKM treatment services 295 (34.0%)	Family doctor		13 (4.4%)	4/9	4	3	0	2	2
	Visit-based treatment		51 (17.3%)	0/51	41	45	0	3	4
	Ordinary treatment		231 (78.3%)	72/60	2	38	0	0	0
Awareness improvement services (34, 3.9%)	TKM experience		6 (17.7%)	6/0	3	0	0	0	0
	Anti-smoking		25 (73.5%)	25/0	2	1	0	0	0
	School TKM programs		3 (8.8%)	0/3	3	3	1	1	1
Total			867 (100%)	751 (86.6%)/ 116 (13.4%)	203 (23.4%)	171 (19.7%)	8 (0.9%)	22 (2.5%)	15 (1.7%)

**Table 3:** Key examples of TKM services provided by communities.

Types		Examples	
Health promotion	Elderly	(Providing organization)	Community health center
		(Targets)	Elderly
		(Program Name)	Solid bone fall prevention Program
		(Contents)	Fall prevention training and Gigong
		(No. of target customers)	320 per year
		(Program evaluation)	BP, blood glucose, BMI, muscle strength, and balance
		(Satisfaction)	94%
Complex (TKM treatment + health promotion)	Female (pregnant women)	(Providing organization)	Community health center and TKM clinics
		(Targets)	Post-natal mothers
		(Program Name)	Post-natal health support program
		(Contents)	Treatment: Herb medicine (postpartum) Health promotion:
		(No. of target customers)	1000 per year
		(Program evaluation)	Submit a log after providing the service (without a separate evaluation)
		(Satisfaction level)	-
TKM treatment	Visit-based treatment	(Providing organization)	Community health center
		(Targets)	People with limited mobility
		(Program Name)	TKM home-visit
		(Contents)	Treatment (acupuncture, moxibustion, and herb medicine)
		(No. of target customers)	360 per year
		(Program evaluation)	Submit a log after providing the service (without a separate evaluation)
		(Satisfaction level)	Executed (the numbers are not submitted)
Awareness improvement	Anti-smoking	(Providing organization)	TKM clinics
		(Targets)	Smokers
		(Program Name)	(Content)
		(Contents)	Stop smoking treatment and consultation
		(No. of target customers)	120 per year
		(Program evaluation)	BP, blood glucose, BMI, height, health awareness, quality of life, VAS, self-esteem
		(Satisfaction level)	88.8%

## Data Availability

The data will be made available upon reasonable request.
